# Novel Options to Counteract Oral Biofilm Formation: *In Vitro* Evidence

**DOI:** 10.3390/ijerph19138056

**Published:** 2022-06-30

**Authors:** Alessandra Odorici, Bruna Colombari, Pierantonio Bellini, Aida Meto, Irene Venturelli, Elisabetta Blasi

**Affiliations:** 1 School of Doctorate in Clinical and Experimental Medicine, Laboratory of Microbiology and Virology, University of Modena and Reggio Emilia, Via G. Campi 287, 41125 Modena, Italy; odorici.alessandra@gmail.com; 2 Department of Surgery, Medicine, Dentistry and Morphological Sciences with Interest in Transplant, Oncology and Regenerative Medicine, Laboratory of Microbiology and Virology, University of Modena and Reggio Emilia, Via G. Campi 287, 41125 Modena, Italy; bruna.colombari@unimore.it (B.C.); pierantonio.bellini@unimore.it (P.B.); 3 Department of Dentistry, Faculty of Dental Sciences, University of Aldent, 1007 Tirana, Albania; 4 Department of Dentistry, Faculty of Dental Medicine, University of Western Balkans, 1051 Tirana, Albania; 5 School of Specialization in Microbiology and Virology, University of Modena and Reggio Emilia, Via G. Campi 287, 41125 Modena, Italy; 165721@studenti.unimore.it

**Keywords:** microbial biofilm, biomimetic hydroxyapatite, MicroRepair, pomegranate extract, antimicrobial compounds

## Abstract

Biofilm production on biotic and abiotic surfaces is crucial in the pathogenesis of most infections, particularly those occurring in the oral cavity. Its prevention and/or control may greatly facilitate the management of patients with oral diseases. Here, the antibiofilm activity of a biomimetic hydroxyapatite and a natural compound, MicroRepair (MicroR) and pomegranate (PomeGr), respectively, was assessed. By luminescence/fluorescence-based assays, *Pseudomonas aeruginosa* (*P. aeruginosa*), *Staphylococcus aureus* (*S. aureus*) and *Candida albicans* (*C. albicans*) were tested for biofilm production in the presence of MicroR and/or PomeGr. We found that both MicroR and PomeGr could affected biofilm production; however, the efficacy of the two, given alone or in combination, varied according to the microbial agent considered. These data open to clinical studies aimed at defining the most efficacious protocols to counteract oral biofilm-associated infections.

## 1. Introduction

The oral cavity is described as a highly complex habitat, involving resident microbial communities that play a key role in local homeostasis. Various parameters, such as poor oral hygiene, dental appliances, diet, drugs, systemic diseases, etc., are also known to influence a health condition [1]. Alterations in oral homeostasis may allow opportunistic pathogens to locally predominate and then organize themselves into microbial biofilms. This homeostatic imbalance leads to frequent oral diseases, such as caries, periodontitis, mucositis, etc. [1].

MicroRepair (MicroR) is a recently described biomimetic compound made of carbonate-hydroxyapatite-zinc crystals; thanks to its biomimetic properties, it is able to interact with tooth hydroxyapatite, favoring enamel remineralization [2]. Moreover, due to its presence in Zn^2+^ ions, MicroR is expected to mediate direct antimicrobial effects. Accordingly, an *in vitro* study has recently demonstrated the efficacy of MicroR to counteract the adhesion and persistence of oral bacteria onto orthodontic elastics [3].

The dramatic increase in drug-resistance, mostly due to improper use of antibiotics [4,5], has encouraged research into alternative drugs, such as natural compounds, including in the field of dentistry. In this respect, antimicrobial agents such as herbs, spices, and probiotics demonstrate effective properties against dental diseases [6]. In particular, pomegranate, the fruit of *Punica granatum* L. (PomeGr), has received significant attention due to its high content of phenolic compounds, likely mediating antimicrobial effects against oral pathogens [7,8].

*Pseudomonas aeruginosa* (*P. aeruginosa*), *Staphylococcus aureus* (*S. aureus*) and *Candida albicans* (*C. albicans*) are opportunistic pathogens responsible of different clinical conditions involving the oral cavity [9]. Due to their strong ability to adhere and persist on abiotic and biotic surfaces, they often cause biofilm-associated infections, which are clinically relevant and difficult-to-treat [10]. In particular, *P. aeruginosa* is responsible for apical periodontitis, pulp necrosis, pulpitis or mandibular/maxillary alveolitis [11] and its organization as a biofilm causes a 4-fold enhancement in drug resistance [12]. Additionally, the high prevalence of *S. aureus* has been described in plaque and tongue samples from systemically healthy subjects with periodontal health, gingivitis or chronic periodontitis [13]. In addition to being a systemic opportunistic pathogen, *C. albicans* also influence mucosal bacterial microbiome at the oral and intestinal mucosa level [14].

Here, we investigated the effects of MicroR and PomeGr, either alone or in combination, on biofilm production by *P. aeruginosa*, *S. aureus* and *C. albicans*, as widely used prototypes of Gram-negative, Gram-positive and fungal pathogens. Using *in vitro* models employing ductile and sensitive luminescence and fluorescence-based assays [15,16], the antibiofilm activity of MicroR and PomeGr has been established.

## 2. Materials and Methods

### 2.1. Compounds

The biomimetic hydroxyapatite (HA) MicroR and the PomeGr peel extract were supplied by Coswell S.p.A. (Bologna, Italy); the former was a commercially available product, while the latter had been supplied as a suspension containing PomeGr peel extract, Saccharomyces ferment lysate filtrate, citric acid, sodium benzoate and potassium sorbate. The MicroR was sterilized by filtration and the PomeGr was treated by autoclave prior to being used in the assays described below.

### 2.2. Microbial Strains and Growth Condition

The bioluminescent bacterial strains, *P. aeruginosa* (strain P1242) and *S. aureus* (Xen29) and the fluorescent fungal strain (GFP-tagged strain derived from *C. albicans* SC5314) were used. According to previously detailed protocols [11], the viable bacterial or fungal cells constitutively emitted bioluminescent or fluorescent signals that could be recorded and quantified by a Fluoroskan reader (Thermo Fischer Scientific, Waltham, MA, USA). Such values, expressed as Relative Luminescence Units (RLU for bacterial cells) or Relative Fluorescence Units (RFU for fungal cells), allowed us to directly measure the amounts of viable microorganisms present in the control and experimental groups.

Operationally, in line with other studies [16], bacterial and fungal cells from −80 °C glycerol stocks were initially seeded onto Tryptic Soy Agar (TSA) or Sabouraud Dextrose Agar (SAB) (OXOID, Milan, Italy) plates, respectively, and incubated overnight at 37 °C. Then, isolated colonies were collected, added to 10 mL of Tryptic Soy Broth (TSB) or Sabouraud broth (OXOID, Milan, Italy) and allowed to grow overnight at 37 °C under gentle shaking, prior to being used for biofilm production.

### 2.3. Assessment of MicroR and PomeGr Effects on Microbial Biofilm Formation

According to previously described protocols [15,16], overnight bacterial or fungal cultures were diluted by the appropriate medium and seeded in 96 black well-plates (10^6^/mL; 100 µL/well); 100 μL of medium (untreated), MicroR or PomeGr were added to each well (the compounds were tested either alone or in combination, at different doses). Then, the plates were incubated at 37 °C for 24 h. Thereafter, the samples were washed twice with phosphate-buffered saline (PBS) to remove the planktonic cells and the bioluminescence or fluorescent signal/well was measured and expressed as RLU (bacterial biofilm) or RFU (fungal biofilm); these values represented the amounts of biofilm produced, under the different experimental conditions.

### 2.4. Statistical Analysis

The Shapiro–Wilk test was used to analyze the distribution of the data within each experimental group. Differences between groups were analyzed by the Kruskal–Wallis’s test, followed by uncorrected Dunn’s multiple comparisons test. Statistical analysis was performed by using GraphPad Prism 8 software. Values of *p* *≤* 0.05 were considered statistically significant (Family-wise significance and Confidence level, 0.05).

## 3. Results

### Antibiofilm Efficacy of MicroR and/or PomeGr

**Figure 1:**
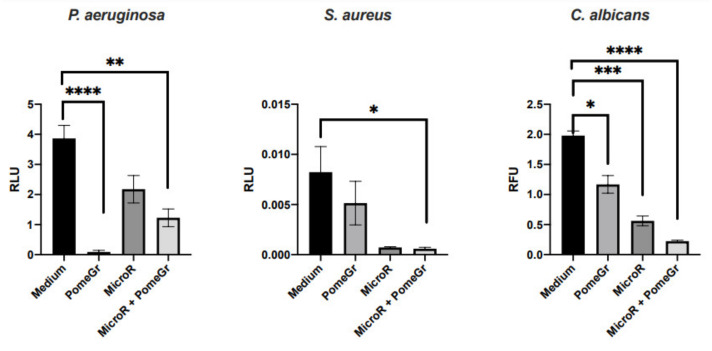
Antibiofilm efficacy of MicroR and/or PomeGr against *P. aeruginosa*, *S. aureus* and *C. albicans*. The microorganisms (10^6^/mL) were exposed to MicroR (4.7 mg/mL) or PomeGr (5.6%) or both and incubated at 37 °C for 24 h; then, biofilm was quantified by luminescence or fluorescence analysis, as detailed elsewhere. The data are the mean ± standard deviation of 6 to 9 determinations from 3 to 4 experiments. Statistical analysis was performed by using GraphPad Prism 8 software. The asterisks indicate statistically significant differences of medium *vs*. treated samples (* *p* = 0.05, ** *p* < 0.01, *** *p* < 0.001 and **** *p* < 0.0001).

In the present study, MicroR and PomeGr, either alone or in combination, have been investigated for their effects against biofilm production by three major prototypes Gram-negative and Gram-positive bacterial cell and fungal cell pathogens, namely *P. aeruginosa*, *S. aureus* and *C. albicans*. Preliminarily, the antimicrobial activity of MicroR and PomeGr was evaluated against planktonic cells, employing the RLU/RFU assay to assess the MicroR and the standard CLSI/NCCLS micro-broth dilution assay to assess the PomeGr. The microbial cells, *P. aeruginosa*, *S. aureus* or *C. albicans,* were exposed to serial dilutions of each compound for 24 h and their Minimal Inhibitory Concentration (MIC) was established. The MIC values were 2.8% (PomeGr) and 2.35 mg/mL (MicroR), irrespective of the microorganism assessed (data not shown). Based on these results, the 2xMIC conditions, namely MicroR at 4.7 mg/mL and PomeGr at 5.6%, were tested hereafter for their effects against biofilm. In particular, *P. aeruginosa*, *S. aureus* and *C. albicans* (10^6^/mL) were exposed to MicroR and/or PomeGr for 24 h at 37 °C in order to allow biofilm formation [14,15]. After washing, the luminescence or fluoresce signals were detected as a measure of biofilm was produced. Figure 1 shows that microbial exposure to PomeGr resulted in a drastic decrease in *P. aeruginosa* biofilm, as assessed by RLU measurement; a partial but non-significant reduction was observed in *S. aureus*, while a significant impairment occurred in *C. albicans* exposed to PomeGr. The MicroR treatment reduced biofilm formation in all the strains, especially in *C. albicans*, where this reduction was highly significant (*p* < 0.0001).

The combination of MicroR and PomeGr significantly reduced biofilm both in *S. aureus* and *C. albicans*, while in *P. aeruginosa*, the RLUs were unexpectedly higher than those obtained with PomeGr alone (Figure 1).

When these data were expressed as a percent of biofilm decrease (Table 1), we found that, upon exposure to PomeGr, *P. aeruginosa* was inhibited down to 98%, while *S. aureus* and *C. albicans* biofilm were reduced to 37.5% and 41%, respectively. When exposed to MicroR alone, *P. aeruginosa*, *S. aureus*, and *C. albicans* showed a biofilm reduction ranging from 43% to 91%; yet, only in *C. albicans* was a decrease found to be significant. Finally, when the strains were exposed to PomeGr plus MicroR, the biofilm was reduced between 68% to 93%, with a partial additive effect observed only in *C. albicans*.

The percent of biofilm decrease (treated and untreated samples) was calculated using the values depicted in Figure 1.

## 4. Discussion

Notoriously, the composition and structure of biomimetic HA are similar to biological systems such as human hard tissues, therefore it is able to integrate into host structures without altering or dissolving its own or neighboring elements. Several studies have shown the possibility of replacing some ions within HA to strengthen its properties; for example, the replacement of Ca^2+^ with Zn^2+^ significantly increases the antimicrobial properties of such biomimetic material [2]. Nowadays, HA is used in dental practice as it has been added to the new formulations of oral hygiene products (mouthwashes, toothpastes, etc.) [2,3]. Here, we provide the first evidence that MicroR, a biomimetic HA, acts as a anti-biofilm agent. In particular, MicroR was capable to reduce *P. aeruginosa* and *S. aureus* biofilm, as measured by the luminescence assay; however, such a decrease did not reach statistical significance. On the other hand, the reduction observed in MicroR-treated *C. albicans* returned a *p* < 0.001; when these variations were expressed as a percentage of biofilm decrease, we obtained 43% for *P. aeruginosa*, 91% for *S. aureus* and 72% for *C. albicans*. Using other doses of MicroR or PomeGr, we failed to detect better results (data not shown).

Furthermore, the combination of the two, MicroR and PomeGr, provided heterogeneous and somehow unexpected results. Indeed, intermediate levels of biofilm inhibition were observed with *P. aeruginosa*, suggesting that MicroR might partially mask the elevated anti-*P. aeruginosa* biofilm efficacy observed with PomeGr alone. Differently, the combination MicroR and PomeGr exerted anti-*S. aureus* effects that reached statistically significant values. Finally, when used together against *C. albicans*, MicroR and PomeGr clearly showed additive effects as inhibitors of fungal biofilm formation. The heterogeneous trends observed with the three microbial agents remain unexplained. Undoubtedly, major differences in the composition and structure of the three microbial agents assessed partially explain the results obtained.

Additionally, we may envisage that MicroR and PomeGr might have differently affected the secretory profile of *P. aeruginosa*, *S. aureus* and *C. albicans*, which are known to release profoundly different sets of crucial molecules, including auto-inducers [15], to produce and establish their biofilm. In any case, by novel *in vitro* assays, we provide the first evidence that microR and PomeGr exert antibiofilm activity and that their action may be additive. Thus, although there are limitations to any *in vitro* model, this work opens the door to further studies on the antibiofilm activity of MicroR and PomeGr against clinical isolates and, even more, multispecies microbial biofilms, including cariogenic bacterial species [17], that commonly afflict the oral cavity.

## 5. Conclusions

By means of an *in vitro* model, the antibiofilm activity of a biomimetic hydroxyapatite, MicroRepair, and a natural compound, pomegranate, has been established. Given the crucial role of biofilm formation in the pathogenesis of many oral diseases, these data offer a basic rationale for the design of trials aimed at maintaining health conditions or recovering homeostatic imbalance in the oral cavity.

## Figures and Tables

**Table 1:** Percent of microbial biofilm decrease upon exposure to PomeGr and/or MicroR.

Treatments	Biofilm Decrease (%)		
	*P. aeruginosa*	*S. aureus*	*C. albicans*
PomeGr	98	37.5	41
MicroR	43	91	72
MicroR + PomeGr	68	93	89

## Data Availability

The data presented in this study are available on request from the corresponding authors.
